# BR deficiency causes increased sensitivity to drought and yield penalty in cotton

**DOI:** 10.1186/s12870-019-1832-9

**Published:** 2019-05-28

**Authors:** Eryong Chen, Xueyan Zhang, Zuoren Yang, Chaojun Zhang, Xiaoqian Wang, Xiaoyang Ge, Fuguang Li

**Affiliations:** 1 Zhengzhou Research base, State Key Laboratory of Cotton Biology, Zhengzhou University, Zhengzhou, 450000 China; 2 State Key Laboratory of Cotton Biology, Institute of Cotton Research, Chinese Academy of Agricultural Sciences, Anyang, 455000 China

**Keywords:** ABA, Brassinosteroid (BR), Cotton (*Gossypium hirsutum* L.), Drought stress, *pag1*, Proteomic

## Abstract

**Background:**

Brassinosteroids (BRs) play crucial roles in drought tolerance, but the underlying molecular mechanisms remain unclear in the important oilseed and fiber crop, cotton (*Gossypium hirsutum* L.).

**Results:**

To elucidate how BRs mediate drought tolerance in cotton, a cotton brassinosteroid (BR)-deficient mutant, *pag1* (*pagoda1*), was employed for analysis. Importantly, the *pag1* mutant showed increased sensitivity to drought stress, with shorter primary roots and fewer lateral roots. The number of stomata was significantly increased in the mutant, and the stomata aperture was much wider than that of the control plants. These mutant plants therefore showed an increased water loss rate. Furthermore, the abscisic acid (ABA) content, photosynthetic efficiency and starch content of the mutant were significantly lower than those of the wild type. The overall performance of the mutant plants was worse than that of the wild-type control under both normal and drought conditions. Moreover, Proteomic analysis revealed reduced levels of stress-related proteins in *pag1* plants.

**Conclusions:**

These results suggest that BRs may modulate the drought tolerance of cotton by regulating much genes that related to drought stress and multiple organ responses to drought, including root growth, stomata development, the stomata aperture and photosynthesis. This study provides an important basis for understanding drought resistance regulated by BRs and cultivating drought-resistant cotton lines.

**Electronic supplementary material:**

The online version of this article (10.1186/s12870-019-1832-9) contains supplementary material, which is available to authorized users.

## Background

Brassinosteroids (BRs), a group of plant-specific polyhydroxylated steroidal hormones were first isolated from *B. napus* pollens [1] and are regarded as essential in regulating multiple processes during normal plant growth and development, including cell division and elongation, vascular differentiation, root development, guard cell development, reproductive processes, and responses to various biotic and abiotic stresses [2–4]. The levels of bioactive endogenous BRs in plants are regulated by both biosynthesis and catabolism. CYP734A1/BAS1, a cytochrome P450 monooxygenase, catalyzes the C-26 hydroxylation of castasterone (CS) and brassinolide (BL), which are active BRs [5, 6]. *PAG1* is the cotton homolog of *CYP734A1/BAS1* and encodes a cytochrome P450 monooxygenase. The *pag1* mutant shows reduced fiber elongation, in addition to the typical phenotypes of BR mutants, such as dwarfed stature and dark green leaves [7].

The function of BRs in stress tolerance has been frequently reported. A study by Kagale showed that 24-*epi*-BL (24-*epi*brassinolide) treatment could enhance drought tolerance in both *Arabidopsis thaliana* and *B. napus* seedlings [8]. In maize, BL application improves drought tolerance by modulating enzymatic antioxidants and the leaf gas exchange system [9]. Another study demonstrated that BRs could enhance tolerance to oxidative stress induced by polyethylene glycol (PEG) treatment through BR-induced NO production and NO-activated ABA biosynthesis in maize leaves [10]. In cucumber, elevated H_2_O_2_ levels due to enhanced NADPH oxidase activity are involved in BR-induced tolerance to stresses, such as photo-oxidative and cold stress and *Cucumber mosaic virus* infection [11]. The *Arabidopsis* BR-deficient mutant *det2–1* was found to be more sensitive to salt stress than wild-type plants [12]. Moreover, overexpression of the BR biosynthesis gene *AtDWF4* in *Arabidopsis* increases the cold tolerance of transgenic seedlings [13]. One of the mechanisms through which BRs participate in cold tolerance might be regulation of pectin methylesterase (PME) activity [14]. In addition, overexpression of *AtDWF4* in *B. napus* simultaneously increases seed yield and stress tolerance, and transcriptome analysis supported the integrated effects of BRs on growth and stress responses [15]. In tomato, the BR-deficient mutant Micro-Tom shows hypersensitivity to drought stress [16].

Drought is one of the most important abiotic factors that seriously threatens modern agriculture worldwide by limiting plant growth and ecosystem production [17]. As an important renewable resource, cotton ranks second among oilseed crops and is the world’s leading natural fiber crop; it is grown in more than seventy countries worldwide, and its production plays a vital role in the global economy. However, the productivity of cotton is adversely affected by a variety of biotic and abiotic stresses, including drought stress. Therefore, the importance things for enhancing cotton production are investigating molecular mechanisms of cotton stress tolerance and cultivating new drought-resistant cotton varieties [18].

BRs confer tolerance to a wide range of abiotic stresses. However, most studies on the effects of BRs on plant stress responses have been conducted using exogenous BRs, and the mechanisms underlying the functions of BRs in abiotic stresses are still unclear. Thus, additional genetic evidence is required to determine the function of BRs in stress tolerance and reveal their functional mechanisms in abiotic stresses. Here, we performed a detailed evaluation of the drought tolerance of *pag1* to determine the role of BRs in the response of cotton to drought. We found that *pag1* exhibits increased sensitivity to drought stress. In addition, compared with CCRI24 control plants, *pag1* plants have lower CS content, increased stomata number and aperture, increased water loss rate, lower ABA content, reduced primary root length and lateral root numbers, lower expression of *GhPIN2* and *GhLAX3* (which encode polar auxin transport carriers), lower photosynthetic efficiency and starch content, and reduced agronomic traits under both normal and drought conditions. Proteomics analysis of *pag1* and CCRI24 (Chinese cotton research institution 24) plants after PEG6000 treatment indicated that a number of stress-related proteins were down-regulated in the *pag1* mutant. Our study is one of the first to reveal the function of BRs in stress tolerance using a BR-deficient cotton mutant, and our data suggest that BRs play important roles in the cotton drought stress response. These findings provide an important basis for fully understanding the mechanism of drought resistance regulated by BRs and for developing BR-based breeding programs for crop improvement.

## Results

### The *pag1* mutant is specifically defective in CS, an active BR

In *Arabidopsis*, *BAS1* (*CYP734A1*) inactivates CS and BL which all belongs to BRs via C-26 hydroxylation [6], and *PAG1* is closely related to *BAS1* [7], suggesting that *PAG1* might function similarly to *BAS1* in the inactivation of CS and BL. However, the exact function of *PAG1* on CS and BL and whether *pag1* is truly a BR-deficient mutant or not require further analysis. To confirm the role of *PAG1* in regulating CS and BL levels, the CS and BL contents of *pag1* and wild-type CCRI24 plants were analyzed. The results showed that the average content of CS was 0.340 ng/g in CCRI24 plants and 0.204 ng/g in *pag1* plants (Table 1); moreover, the test of significant differences showed that the contents of CS were significantly lower in *pag1* plants than in CCRI24 plants (Table 1). Although the content of BL was slightly lower in *pag1* plants than in CCRI24 plants, there was no significant difference in BL contents between the two lines (Table 1). These results suggest that *pag1* is a BR-deficient mutant and that *PAG1* primarily functions in the inactivation of CS.

**Table 1:**
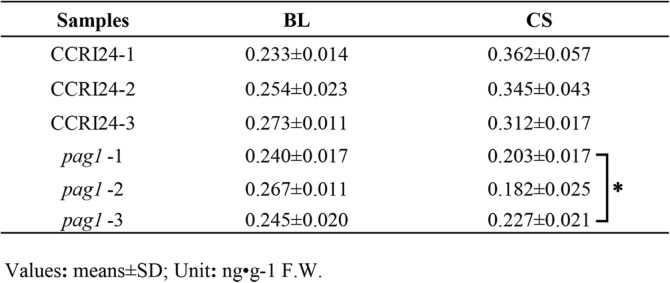
Brassinosteroids (BRs) levels in the *pag1* mutant and CCRI24

Student’s *t* test: *, *P* < 0.05

**Fig. 1:**
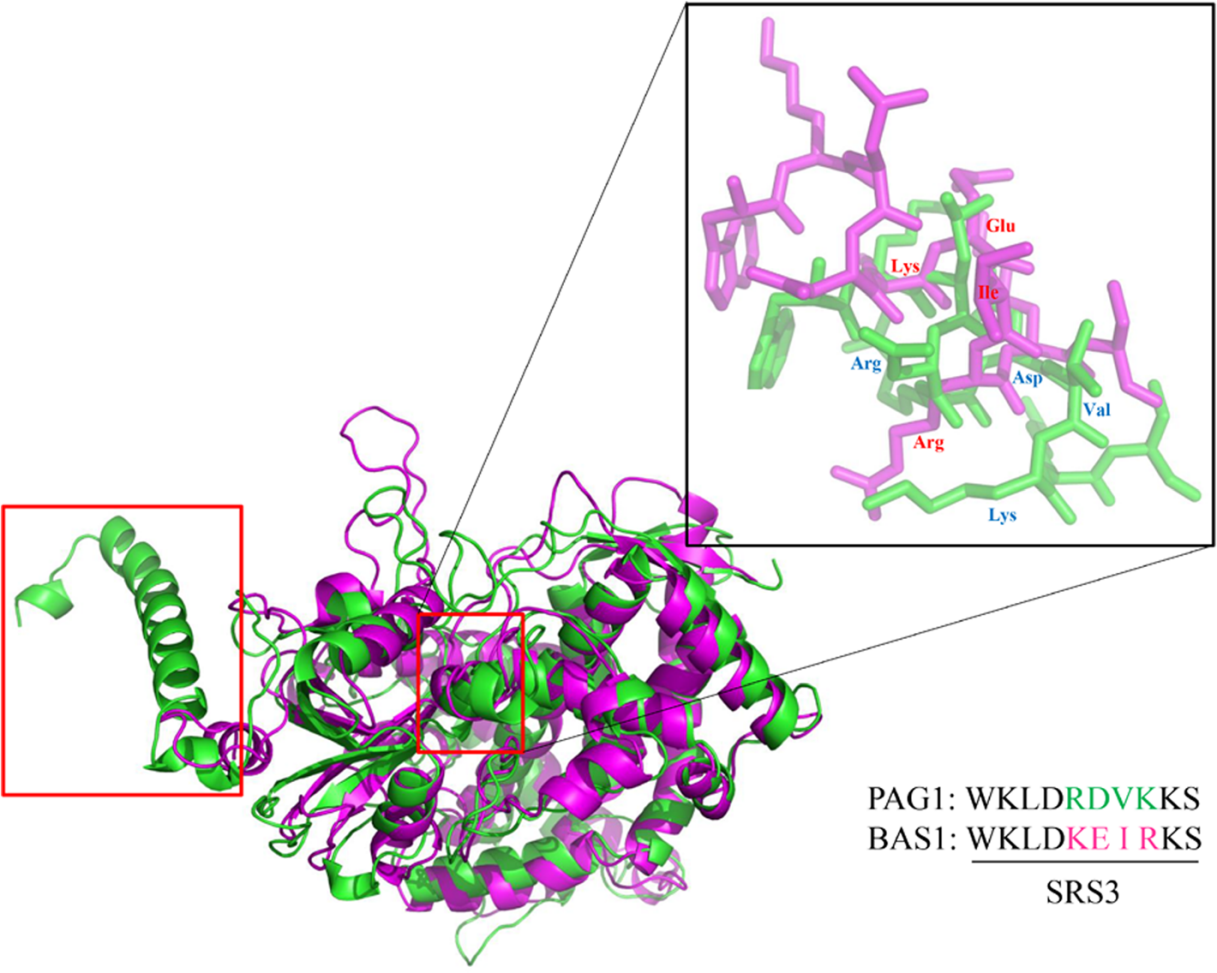
Structural alignment of PAG1 (green) with BAS1 (magenta). The red box indicates the areas of differences in the structures of PAG1 and BAS1. The inset shows the difference in the third substrate recognition site (SRS3) between PAG1 and BAS1

To analyze the specific function of PAG1 in cotton, the well-modeled PAG1 and BAS1 structures all built with Cytochrome P450 4B1 template (PDB ID: 6c93) were applied using the Swiss-Model server [19], then structural alignment of PAG1 with BAS1 was done and shown in Fig. 1. The results of structural analysis showed that there was an extra α-helix at the N-terminus of PAG1, and the third substrate recognition sites (SRS3) of PAG1 and BAS1 did not match perfectly (Fig. 1). The structural differences between PAG1 and BAS1 might be the reason that PAG1 mainly inactivated CS in cotton.

### The *pag1* mutant is sensitive to drought stress

**Fig. 2:**
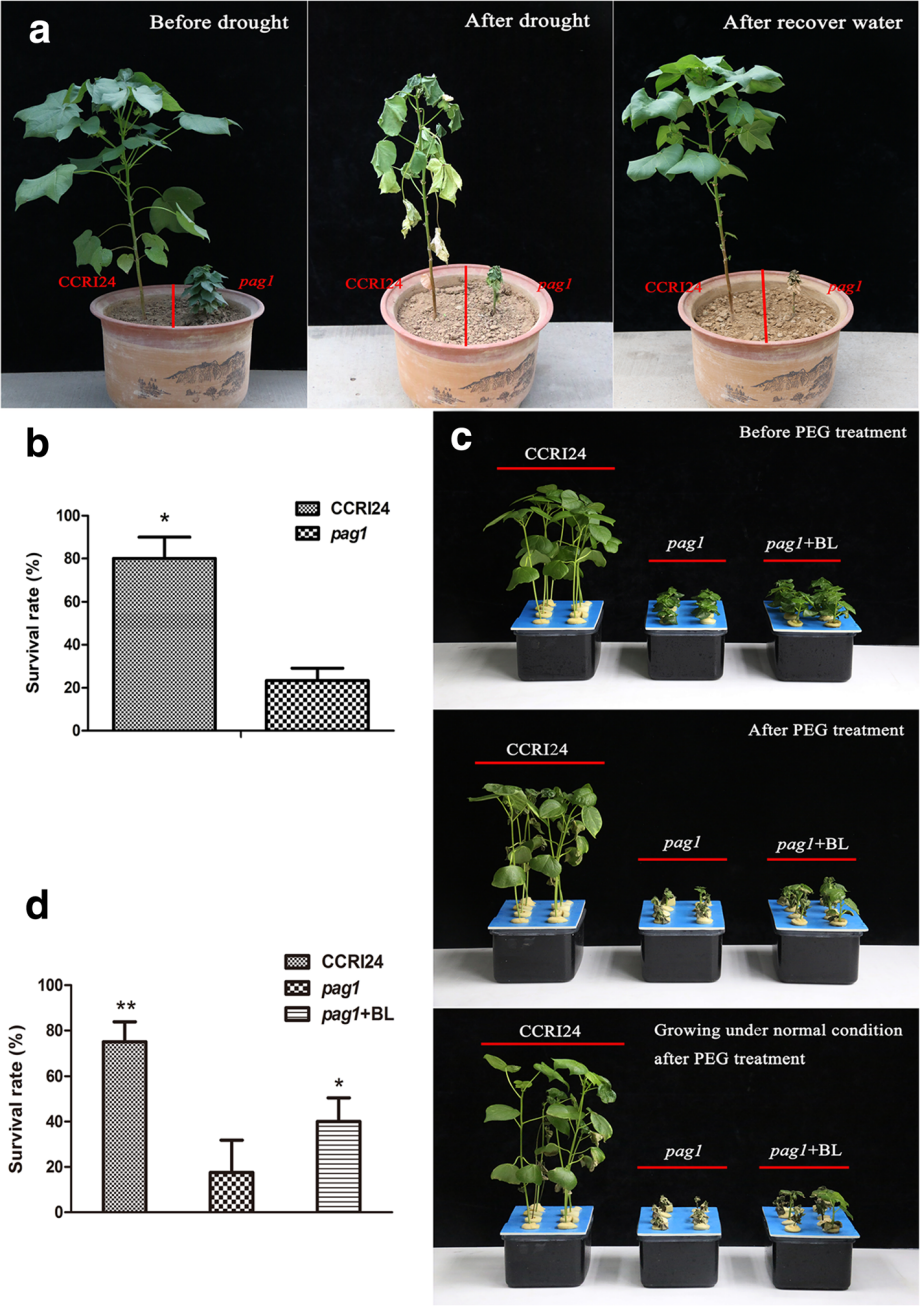
Response of *pag1* and CCRI24 to drought stress. **a** Phenotypes of *pag1* and CCRI24 before drought stress, after drought stress and after water recovery while growing in soil. **b** Analysis of the survival rate after water recovery of *pag1* and CCRI24, as shown in **a**. **c** Phenotype of *pag1* and CCRI24 growing in cotton nutrient solution before PEG treatment, after PEG treatment and growing under normal conditions after PEG treatment. **d** Analysis of the survival rate *pag1* and CCRI24 growing under normal conditions after PEG treatment as shown in **c**. (*t* test): *, *P* < 0.05; **, *P* < 0.01

To evaluate the function of BRs in drought tolerance in cotton, the *pag1* mutant and CCRI24 controls were grown for 50 days in pots, and then watering was stopped. After 25 days, all the leaves of *pag1* plants were wilted, but majority of the CCRI24 leaves remained vigorous (Fig. 2a). After one week of water recovery, the CCRI24 plants resumed normal growth, but the *pag1* plants had died (Fig. 2a), and the statistical result showed that CCRI24 plants had a significantly higher survival rate than *pag1* plants (Fig. 2b).

To determine whether BRs could restore the ability of *pag1* to resist drought stress, CCRI24 plants, *pag1* plants, and *pag1* plants treated with 24-*epi*-BL were grown in hydroculture, and drought stress was simulated by treating three-week-old seedlings with 6% PEG6000. After one week of treatment, a majority of the *pag1* plants were dead, and only 13.3% survived (Fig. 2c, d). The CCRI24 line exhibited a significantly higher survival ratio of 73.3%, and the 24-*epi*-BL-treated *pag1* plants also exhibited a higher survival ratio of 26.7% (Fig. 2d). These results demonstrated that *PAG1* regulates cotton drought tolerance partially by affecting BR levels.

### Increased leaf stomata density and wider stomata aperture in *pag1*

**Fig. 3:**
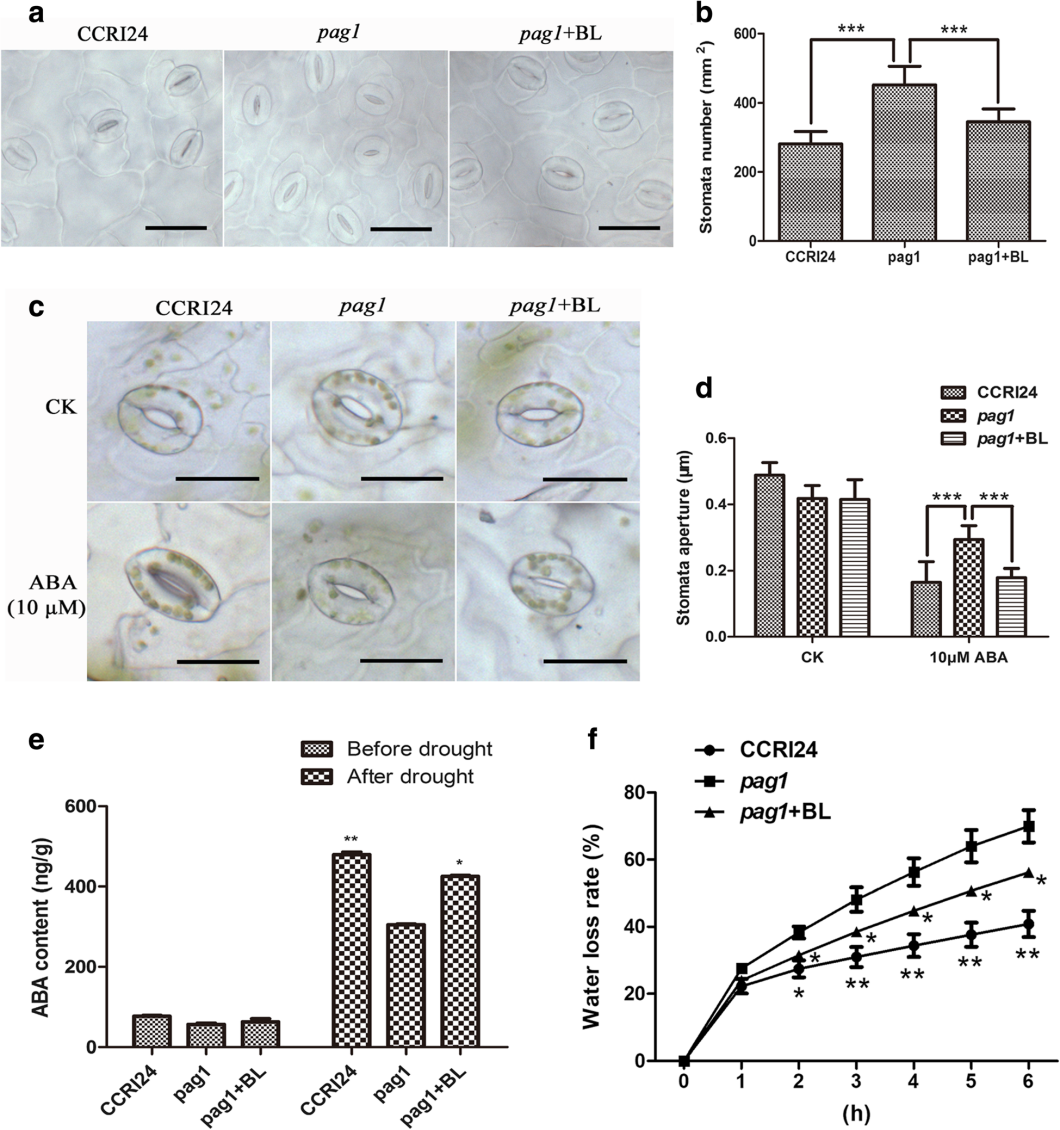
Stomata analysis of *pag1* and CCRI24. **a** Observations of the stomata of *pag1*, CCRI24 and 24-*epi*-BL-treated *pag1* plants at the three-leaf stage. Bar = 100 μm. **b** Stomata number analysis of the *pag1*, CCRI24 and 24-*epi*-BL-treated *pag1* plants shown in **a**. **c** Observations of stomata aperture of *pag1*, CCRI24 and 24-*epi*-BL-treated *pag1* plants at the three-leaf stage under normal conditions and after ABA treatment. Bar = 50 μm. **d** Stomata aperture analysis of the *pag1*, CCRI24 and 24-*epi*-BL-treated *pag1* plants shown in **c**. **e** ABA contents of *pag1*, CCRI24 and 24-epi-BL-treated *pag1* plants under normal conditions and after drought treatment. **f** Water loss rate analysis of *pag1*, CCRI24 and 24-epi-BL-treated *pag1* plants at the three-leaf stage. Student *t* test: *, *P* < 0.05; **, *P* < 0.01; ***, *P* < 0.001

Stomata density and stomata movement (opening and closing) can regulate transpirational water loss, which is a key determinant of drought tolerance [20, 21]. To examine why *pag1* is sensitive to drought stress, the stomata density of *pag1* plants, 24-*epi*-BL-treated *pag1* plants and CCRI24 plants was analyzed at the one-month-old seedling stage. The results showed that the average stomata density in the top fourth leaf of *pag1* plants was 37.8% higher than in CCRI24 plants (Fig. 3a, b), and the stomata density of the 24-*epi*-BL-treated *pag1* leaves was 23.7% lower than that of untreated *pag1* plants (Fig. 3a, b).

ABA modulates stomata closure by guard cells to avoid water loss during drought stress [22]. As shown in Fig. 3c and d, ABA treatment caused more pronounced stomata closure in CCRI24 and 24-*epi*-BL-treated *pag1* leaves than in *pag1* leaves. ABA content analysis showed that the ABA content in *pag1* plants was not significantly different than in CCRI24 and 24-*epi*-BL-treated *pag1* under normal conditions, but the ABA content was significantly lower in *pag1* plants than in CCRI24 and 24-*epi*-BL-treated *pag1* plants after drought treatment, even though ABA contents were increased in all three groups (Fig. 3e). These results demonstrate that BRs regulate the stomata aperture by regulating the ABA content in cotton.

Moreover, a water loss rate assay demonstrated that the water loss rate was significantly lower in both 24-*epi*-BL-treated *pag1* plants and CCRI24 plants than in *pag1* plants (Fig. 3f), and the water loss rate in the 24-*epi*-BL-treated *pag1* plants was also higher than in the CCRI24 plants. These results were consistent with the results of the stomata density and stomata aperture analyses.

### Altered root architecture resulting from lower expression levels of auxin polar transport genes in *pag1*

**Fig. 4:**
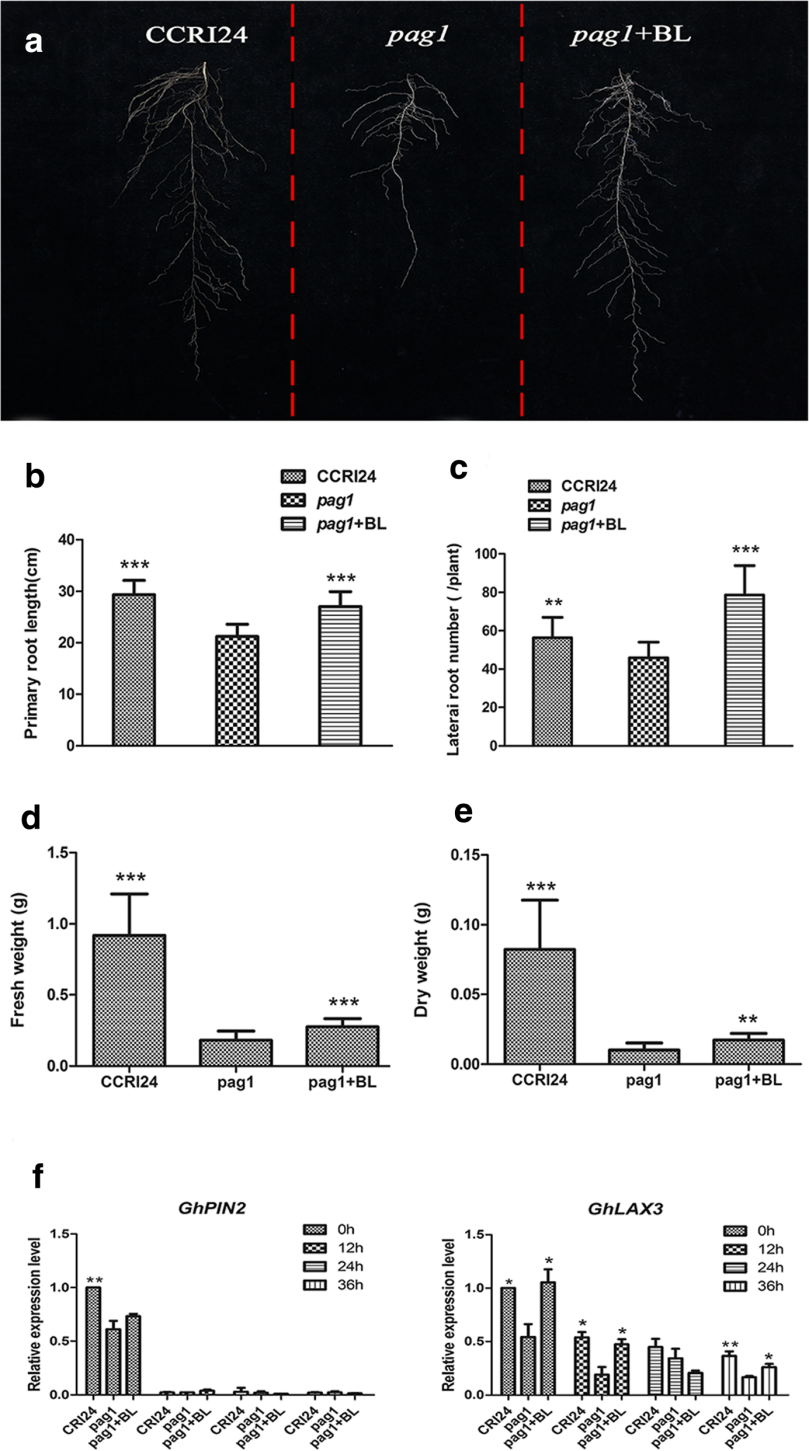
Root phenotype of *pag1* and CCRI24. **a** Root phenotype of *pag1* plants, CCRI24 plants and *pag1* plants treated with 24-*epi*-BL at the three-leaf stage. **b** Primary root length analysis of the *pag1*, CCRI24 and 24-*epi*-BL-treated *pag1* plants shown in **a**. **c** Lateral root number analysis of the *pag1*, CCRI24 and 24-*epi*-BL-treated *pag1* plants shown in **a**. **d** Root fresh weight of *pag1*, CCRI24 and 24-*epi*-BL-treated *pag1* plants at the three-leaf stage. **e** Root dry weight of *pag1*, CCRI24 and 24-*epi*-BL-treated *pag1* plants at the three-leaf stage. **f** Expression levels of *GhPIN2* and *GhLAX3* in *pag1*, CCRI24 and 24-*epi*-BL-treated *pag1* plants at the three-leaf stage under normal conditions and at 12 h, 24 h and 36 h after PEG treatment. (*t* test): *, P < 0.05; **, P < 0.01; ***, P < 0.001

Deep root systems help to increase the water accessibility of plants. *pag1* plants grown in hydroculture exhibited shorter primary root lengths and fewer lateral roots than CCRI24 plants at the trefoil stage; moreover, the primary root length and lateral root number were restored when *pag1* plants were grown in a nutrient solution containing 10 nM 24-*epi*-BL (Fig. 4a, b, c). The root biomass of one-month-old *pag1* seedlings was markedly decreased, whereas the root biomass of *pag1* seedlings grown in a nutrient solution containing 10 nM 24-*epi*-BL was higher, but still lower than that of CCRI24 seedlings (Fig. 4d). Consequently, the root dry weight of *pag1* seedlings was significantly lower than that of CCRI24 seedlings, and the root dry weight of 24-*epi*-BL-treated *pag1* seedlings was considerably higher than that of untreated *pag1* seedlings (Fig. 4e). The altered root architecture of *pag1* plants might negatively contribute to drought tolerance, as shorter primary roots and fewer lateral roots could result in reduced water and nutrient uptake.

Auxin is an important phytohormone that regulates root growth; thus, we speculated that BRs could promote the expression of auxin polar transport genes in plant roots [23]. Auxin polar transport requires auxin polar transport carriers, and qPCR was performed to analyze the gene expression levels of two auxin polar transport carrier genes, *GhPIN2* and *GhLAX3*. The expression level of *GhPIN2* was significantly lower in *pag1* plants than in CCRI24 and 24-*epi*-BL-treated *pag1* plants under normal conditions, but smaller differences were observed among *pag1*, CCRI24 and 24-*epi*-BL-treated *pag1* plants after 12 h, 24 h, and 36 h of PEG6000 treatment (Fig. 4f). The expression of *GhLAX3* was significantly lower in *pag1* plants than in CCRI24 plants, both under normal conditions and after treatment with PEG6000; in addition, its expression was lower in *pag1* plants than in 24-*epi*-BL-treated *pag1* plants under the same conditions described above, except in plants treated with PEG6000 for 24 h (Fig. 4f). These results suggested that BRs regulate the expression of auxin polar transport genes to affect auxin polar transport and, in turn, regulate cotton root growth.

### Reduced photosynthetic rate and starch content in the *pag1* mutant

**Fig. 5:**
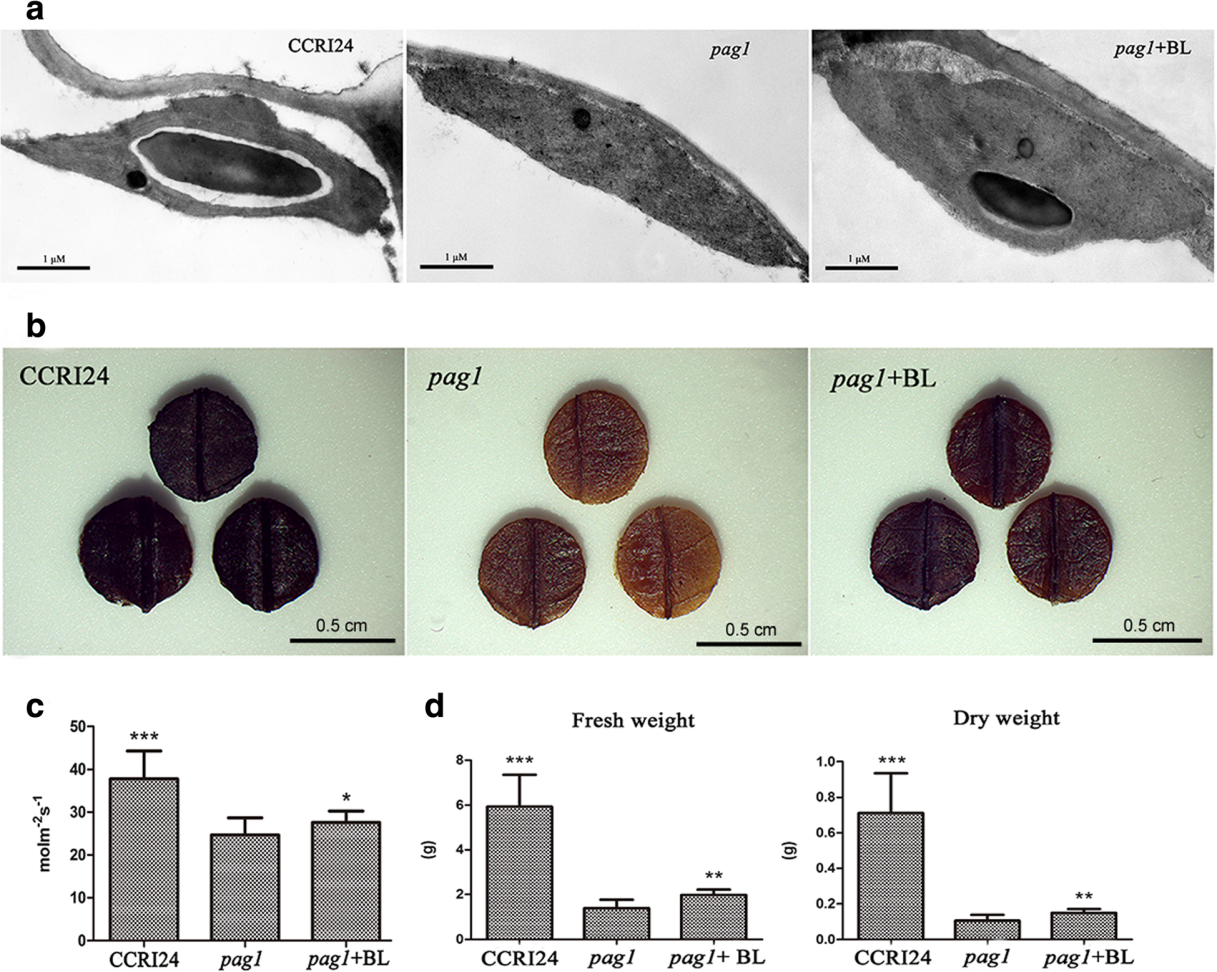
Analysis of photosynthetic rates and starch contents in *pag1* and CCRI24. **a** Observations of starch granules in the top fourth leaves of *pag1*, CCRI24 and 24-*epi*-BL-treated *pag1* plants using transmission electron microscopy. Bar = 1 μm. **b** Observations of starch contents in the top fourth leaves of *pag1*, CCRI24 and 24-*epi*-BL-treated *pag1* plants using the iodine staining method. Bar = 0.5 cm. **c** Photosynthetic rate analysis of *pag1*, CCRI24 and 24-*epi*-BL-treated *pag1* plants. **d** Fresh weight and dry weight of *pag1*, CCRI24 and 24-*epi*-BL-treated *pag1* plants at the three-leaf stage. (*t* test): *, *P* < 0.05; **, *P* < 0.01; ***, *P* < 0.001

Studies have shown that BRs can promote the plant photosynthesis rate [24, 25]. During photosynthesis, fixed carbon is converted to starch in the leaves of most plant species; subsequently, starch accumulates within chloroplasts [26]. In our study, transmission electron microscopy images of sections of the top fourth leaves showed that CCRI24 plants, which contained large starch granules, exhibited a higher starch content than *pag1* plants, which contained almost no starch in their chloroplasts (Fig. 5a). When *pag1* leaves were treated with 24-*epi*-BL, starch deposits were observed in chloroplasts, but these deposits were smaller than those in CCRI24 leaves (Fig. 5a). Iodine staining was also used to detect starch reserves in the leaves of CCRI24, *pag1* and 24-*epi*-BL-treated *pag1* plants. Upon staining, CCRI24 leaves were found to harbor deep blue starch granules, while the staining in *pag1* leaves was lighter. However, 24-*epi*-BL-treated *pag1* leaves exhibited an intermediate blue shade after granule staining, which was deeper in color than that in *pag1* leaves and slightly lighter than that in CCRI24 leaves (Fig. 5b). We also measured photosynthesis in *pag1* and CCRI24 plants at the reproductive stage in the field and found that the photosynthetic rate was reduced by 34.7% in the *pag1* plants compared with CCRI24 plants (Fig. 5c), while the photosynthetic rate of 24-*epi*-BL-treated *pag1* plants was significantly higher than that of *pag1* plants. The leaf photosynthesis rate is positively correlated with the total biomass of a plant [27]. Therefore, the seedling biomass was also examined. Although the fresh and dry weights of CCRI24 plants and 24-*epi*-BL-treated *pag1* plants were significantly higher than those of *pag1* plants, the biomass of 24-*epi*-BL-treated *pag1* plants was still lower than that of CCRI24 plants (Fig. 5d).

### Agronomic performance and cotton yield of the *pag1* mutant in the field

Yield is an important agronomic trait in analyzing stress tolerance of crops [28, 29]. To evaluate the agronomic traits of the *pag1* mutant in the field, *pag1*, CCRI24 and 24-*epi*-BL-treated *pag1* plants were grown in the field in Anyang, Henan Province, China. To evaluate field traits under drought conditions, *pag1*, 24-*epi*-BL-treated *pag1* and CCRI24 plants were grown in an open green house in the same place to simulate normal field conditions, with the exception of rain. As shown in Table 2, under normal conditions, the *pag1* plants showed significantly decreased height, fruit branch number, boll number per plant and seed cotton yield per plant compared with those of the CCRI24 and 24-*epi*-BL-treated *pag1* plants. Under drought conditions, the seed cotton yield and other agronomic traits mentioned above were distinctly decreased in *pag1* plants compared with CCRI24 and 24-*epi*-BL-treated *pag1* plants (Table 2). These results suggest that BR deficiency has a negative effect on agronomic traits and cotton yield under both normal and drought stress conditions in the field. More importantly, under drought conditions, the relative reduction of boll number was much lower in CCRI24 and 24-*epi*-BL-treated *pag1* plants than that in *pag1* plants (Table 2). Besides, the relative reduction of seed cotton yield was much higher in *pag1* plants than that in CCRI24 plants, and also high in *pag1* plants than that in 24-*epi*-BL-treated *pag1* plants though there was no significant difference between them (Table 2). These results suggest that BR deficiency results in easier reduction of yield traits of cotton under drought conditions.

**Table 2:**
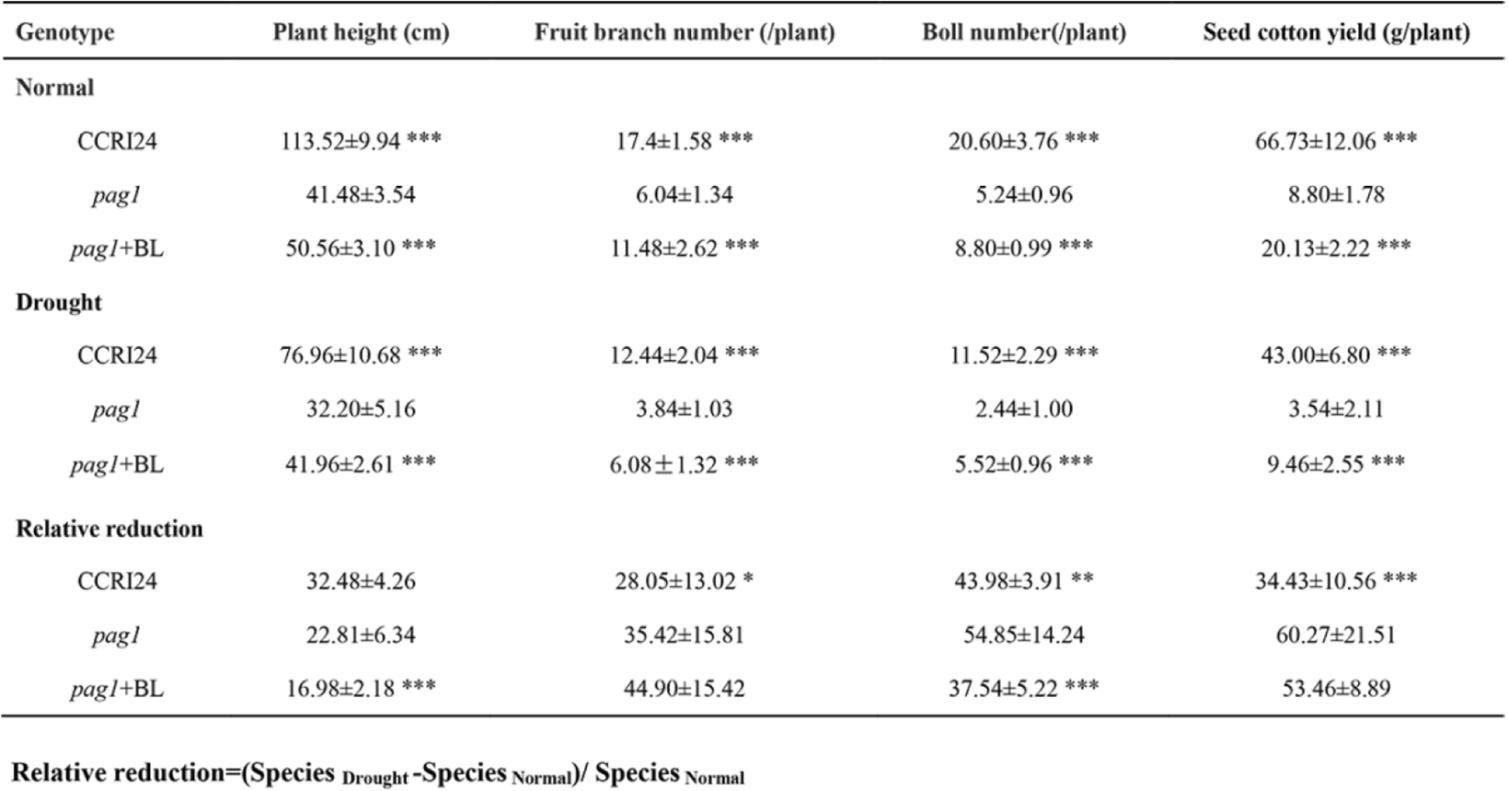
Agronomic traits and cotton yields of *pag1*, CCRI24 and 24-*epi*-BL-treated *pag1* under two below conditions

Student’s *t* test: *, *P* < 0.05; **, *P* < 0.01; ***, *P* < 0.001

### Proteomics reveals numerous proteins involved in the *pag1* response to drought stress

**Fig. 6:**
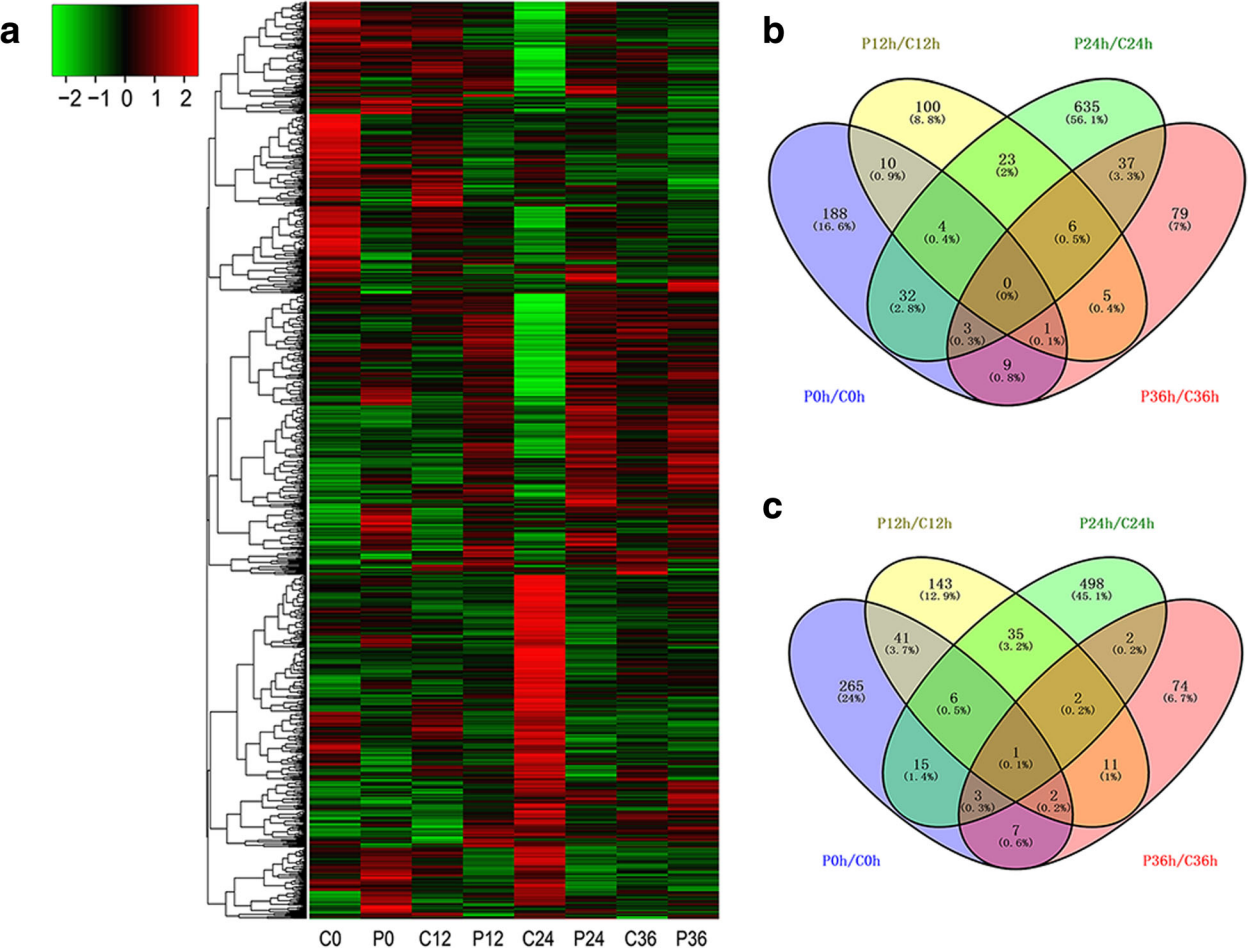
Overview of serial analysis of differentially abundant proteins between *pag1* and CCRI24 after PEG treatment. **a** Hierarchical clustering analysis of all differentially abundant proteins based on iTRAQ data. **b** and **c** Venn diagrams of up- and down-regulated proteins in comparisons of P0/C0, P12/C12, P24/C24 and P36/C36. P, *pag1*; C, CCRI24; 0, 12, 24 and 36 represent the trefoil-stage roots of *pag1* and CCRI24 after treatment with 6% PEG6000 for 0 h, 12 h, 24 h and 36 h, respectively

To explore the underlying molecular mechanisms of BR mediate drought tolerance in cotton, iTRAQ analysis was used to identify differentially abundant proteins in *pag1* and CCRI24 roots, both under normal conditions and after treatment with 6% PEG6000. Under normal conditions, 587 proteins were up- or down-regulated by at least 1.2-fold in *pag1* roots compared with CCRI24 roots. Moreover, at the three time points of PEG6000 treatment (12 h, 24 h and 36 h), 390, 1302 and 242 proteins with a greater than ±1.2-fold difference between *pag1* and CCRI24 roots were identified. In addition, a cluster analysis was performed, and a heatmap was generated to visualize the expression patterns of all differentially abundant proteins in *pag1* and CCRI24 plants (Fig. 6a). Venn diagram results indicated that only a few differentially abundant proteins overlapped between P0h/C0h (*pag1*-0 h/CCRI24-0 h), P12h/C12h (*pag1*-12 h/CCRI24-12 h), P24h/C24h (*pag1*-24 h/CCRI24-24 h) and P36h/C36h (*pag1*-36 h/CCRI24-36 h) (Fig. 6b, c). Among these 1245 down-regulated differentially abundant proteins, only 50 (4.5%), 25 (2.3%) and 13 (1.2%) proteins identified in P0h/C0h overlapped with those identified in P12h/C12h, P24h/C24h and P36h/C36h, respectively (Fig. 6c).

In addition to these differentially abundant proteins, many well-known stress-related genes were found to be down-regulated in *pag1* roots (Table 3). Most of these proteins showed lower expression in *pag1* roots than in CCRI24 roots both under normal conditions and after PEG6000 treatment. These results collectively suggest that the decrease of BRs in *pag1* inhibited the protein abundant of stress-resistance genes under drought stress conditions, thus reducing *pag1* drought tolerance. Among the proteins that were significantly inhibited in *pag1* plants, quite a few were stress-responsive transcription factors, such as MYC2, WRKY25 and WRKY33, indicating that BRs may indirectly regulate the expression of numerous stress-responsive genes. To confirm the iTRAQ results, 10 stress-related genes were selected for qRT-PCR analysis using specific primers (Additional files 6: Table S5). The expression patterns of most of these genes were consistent with the iTRAQ results that the protein levels were lower in *pag1* plants than in CCRI24 plants, with the exception of three genes for which larger differences were observed at different treatment time points (Additional file 1: Figure S1). These results suggest that the iTRAQ results are reliable and useful.

**Table 3:**
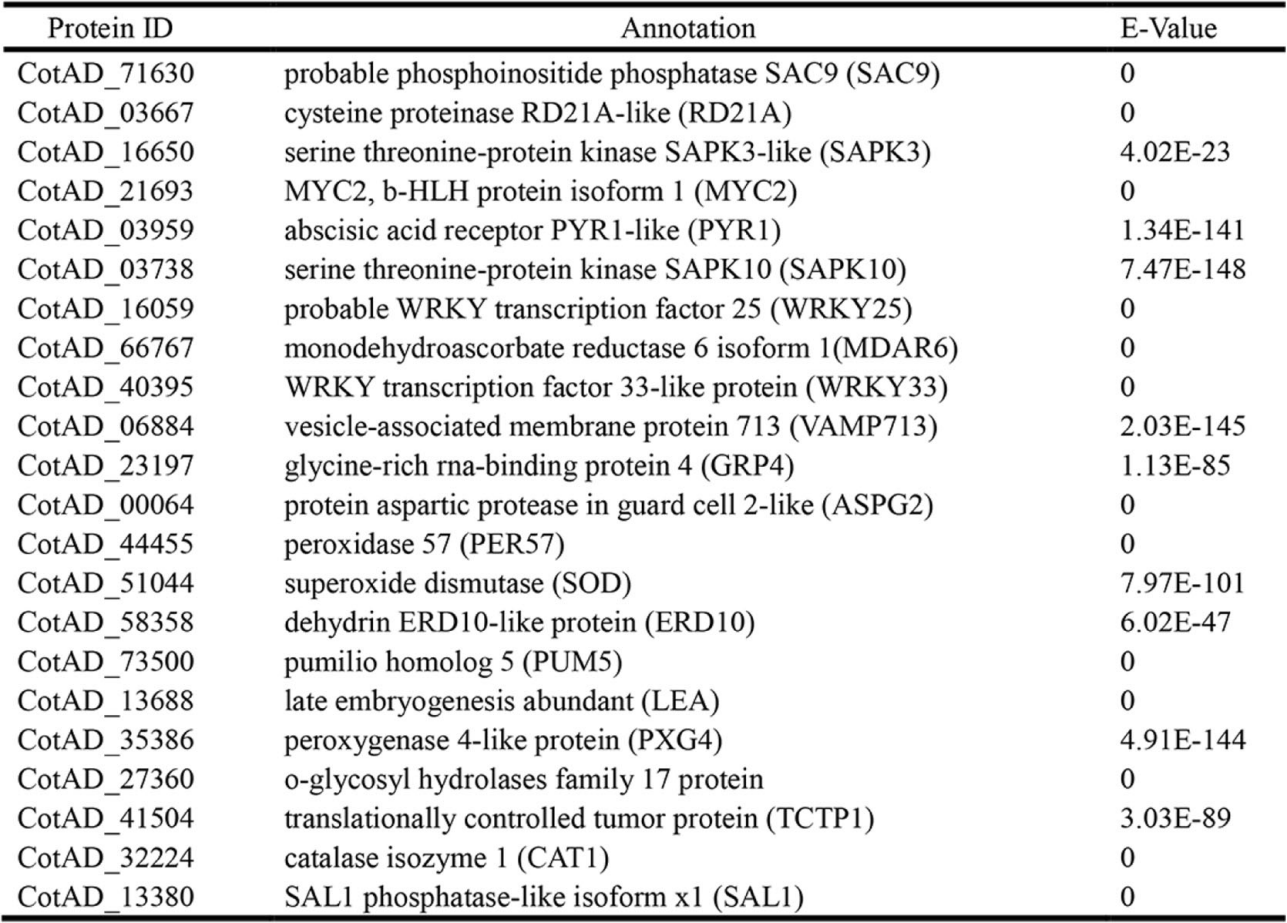
Down-regulated proteins related to stress tolerance in the *pag1* mutant

## Discussion

### The *PAG1* gene mainly affects CS content in cotton

The levels of bioactive endogenous BRs in plants are regulated by both biosynthesis and catabolism [30]. Mutation of *CPD* results in a typical BR-deficient phenotype, including an extremely dwarfed stature, dark green color, delayed flowering, and reduced male fertility [31, 32]. In vitro, biochemical analysis showed that CPD catalyzes the C-3 oxidation of early BR biosynthetic intermediates [33]. Ectopic overexpression of *BAS1*/*CYP734A1*, which encodes a BR-inactivating enzyme, in tobacco results in dwarf plants with dark-green leaves and short stems and petioles [5]. Moreover, the *bas1-D*/*phyB-4* mutant exhibits low levels of BL and CS and accumulates 26-hydroxybrassinolide [5], primarily because the function of BAS1 is to catalyze the C-26 hydroxylation of CS and BL [5, 6]. Although a number of genes that regulate BR content have been identified in *Arabidopsis*, only a few genes have been reported to regulate BR levels in cotton. Here, we report an analysis of the *PAG1* gene, which is a homolog of *BAS1*/*CYP734A1* [7]. The cotton *pag1* mutant, which exhibits high expression of the *PAG1* gene, displays a typical BR-deficient phenotype, including severe dwarfism, dark green leaves and reduced fiber elongation [7]. These observations suggest that PAG1 functions similarly to BAS1 in catabolizing bioactive BRs in cotton. To confirm the characteristics of the *PAG1* gene in cotton, BL and CS levels were determined in *pag1*. Our results showed that the CS level was significantly lower in the *pag1* mutant than in CCRI24 plants; however, the BL level did not differ between *pag1* and CCRI24 plants (Table 1). These results suggest that the main function of PAG1 might be to specifically catalyze the hydroxylation of CS and reduce total BR levels in cotton, and the reduction of an active BR could result in a typical BR-deficient phenotype in cotton. Structural analysis showed that PAG1 harbored an extra α-helix at N-terminus not found in BAS1 and that the third substrate recognition site (SRS3) of the two proteins exhibited a large difference (Fig. 1). Thus, the different functions of PAG1 and BAS1 might be a result of their protein structure differences and may indicate that PAG1 has a special function in inactivating CS in cotton, as homologous genes can have both similar and different functions in different plants.

### BRs regulate stomata development and inhibit stomata closure induced by ABA

Recently, BRs have been found that it could activate the MAPK kinase kinase YDA to inhibit stomata formation in *Arabidopsis* [34–36]. In our study, we found that the stomata densities of CCRI24 and 24-*epi*-BL-treated *pag1* plants were significantly lower than that of *pag1* plants (Fig. 3a, b). This result indicates a similar function of BRs in inhibiting stomata formation in cotton.

ABA is an important phytohormone that influences stomata aperture movement (opening and closing) [37]. BR application has been shown to enhance the drought tolerance of *Arabidopsis* and *B. napus* seedlings, which is accompanied by ABA accumulation [8]. Our stomata aperture analysis indicated that the *pag1* plants have larger stomata aperture than CCRI24 plants, while the stomata aperture of 24-*epi*-BL-treated *pag1* plants was significantly reduced compared with that in untreated mutants (Fig. 3c, d). Moreover, the ABA content was significantly lower in *pag1* plants than in CCRI24 plants and was remarkably elevated in 24-*epi*-BL-treated *pag1* plants (Fig. 3e). These observations suggest that BRs regulate ABA-inducted stomata aperture movement, thereby regulating drought tolerance in cotton. In addition, ABA regulates plant responses by acting as a signaling molecule under normal or stress conditions [38, 39]. Thus, BRs might also affect stress-related genes that are controlled by ABA to regulate cotton drought tolerance (Additional file 2: Table S1).

Stomata density and stomata movement play key roles in regulating the plant leaf transpiration rate [20, 21]. In this study, we found that the water loss rate was significantly higher in *pag1* plants than in CCRI24 and 24-*epi*-BL-treated *pag1* plants (Fig. 3f), which was consistent with the results regarding stomata density and stomata aperture in *pag1* plants (Fig. 3a-d). These observations suggest that BRs regulate stomata density and ABA-inducted stomata closure, which in turn helps regulate the water loss rate of cotton and, thus, controls drought tolerance.

### BRs interact with auxin to regulate root development in cotton

A thick, deep root system enables plants to extract deep soil water and is considered important in determining plant drought tolerance [40]. It has been observed that a concentration of BL (1-100 nM) can promote root elongation in maize [41]. However, other studies indicated that a low concentration of BL (0.05–0.1 nM) can promote root elongation and 1–100 nM BL inhibits root elongation [23, 42]. In our study, we observed shorter primary root lengths in *pag1* plants than in CCRI24 plants. The root length phenotype of *pag1* mutants could be restored to that of CCRI24 plants after treatment with 24-*epi*-BL (Fig. 4a, b). These results suggest that a proper concentration of BRs can promote root elongation in plants and that BRs are required for root elongation. Moreover, it was found that wild-type plants exhibit more lateral roots than BR-deficient mutants [23]. Similarly, we observed fewer lateral roots in *pag1* plants than in CCRI24 plants, although the phenotype could be restored to that of CCRI24 plants after treatment with 24-*epi*-BL (Fig. 4a, c). Our results provide further evidence that BRs regulate lateral root development in plants. Overall, it can be concluded that the function of BRs in root development is conservative.

Polar auxin transport, which results in distribution of auxin, is involved in almost all auxin-mediated plant growth processes, including root growth [43]. In addition, BRs enhance polar auxin transport and endogenous auxin distribution by up-regulating the expression of auxin efflux carriers, such as PIN2 [44]. Our study showed that the expression level of an auxin efflux carrier gene, *GhPIN2*, was significantly lower in *pag1* plants than in CCRI24 plants and was elevated in 24-*epi*-BL-treated *pag1* plants under normal conditions; furthermore, few differences in gene transcript levels were found in the three plant groups after PEG6000 treatment (Fig. 4f). The transcript level of *GhLAX3*, an auxin influx carrier gene, was significantly higher in CCRI24 and 24-*epi*-BL-treated *pag1* plants than in *pag1* plants under both normal and PEG6000 treatment conditions, except in samples of 24-*epi*-BL-treated *pag1* plants that were treated with PEG6000 for 24 h, although the expression of *GhLEA3* was down-regulated after PEG6000 treatment compared with that under normal conditions (Fig. 4f). These results suggest that BRs mediate root development by regulating the auxin distribution, which is affected by genes involved in polar auxin transport, and in turn play an important role in regulating drought tolerance in cotton.

### BR deficiency inhibits the photosynthetic rate and reduces the yield of cotton

A number of studies have focused on the function of BRs in photosynthesis and determining yields. Xia et al. found that BRs could increase the plant photosynthesis rate and growth by positively regulating many photosynthetic enzymes [24]. Another study revealed that BRs could enhance CO_2_ assimilation, increase glucose pools in leaves, and increase the conversion of glucose to starch in seeds [25]. Furthermore, overexpression of *AtDWF4*, an important BR biosynthetic gene in *B. napus* was shown to increase the seed yield [15]. In our study, the *pag1* mutant was found to exhibit a lower photosynthetic rate than CCRI24 and 24-*epi*-BL-treated *pag1* plants (Fig. 5c). The biomass of *pag1* plants was also lower than that of CCRI24 and 24-*epi*-BL-treated *pag1* plants (Fig. 5d). In addition, the starch content was lower in *pag1* plants than in the other two plant groups (Fig. 5a, b). Moreover, under both normal and drought conditions, the yield of *pag1* plants was lower than that of CCRI24 plants and significantly increased following treatment with 24-*epi*-BL (Table 2). These results revealed that BR deficiency inhibits photosynthesis in cotton and reduces energy accumulation and cotton yield.

Photosynthesis plays a vital role in sucrose and starch synthesis, and sugars such as sucrose, glucose and fructose are involved in drought signaling pathways [45, 46]. The carbohydrate status in drought-stressed plants depends not only on the efficiency of the photosynthetic carbon reduction cycle and sucrose/starch synthesis but also on osmotic adjustment [47–49]. In our research, the *pag1* mutant was found to be more sensitive to drought stress than control plants (Fig. 2a, b). Moreover, the relative reduction of yield traits (boll number and seed cotton yield) observed under drought conditions was much greater in *pag1* plants than in CCRI24 plants and 24-*epi*-BL-treated *pag1* plants, except for the seed cotton yield in *pag1* plants treated with 24-*epi*-BL (Table 2). Thus, all the above results suggested that BR deficiency inhibits energy accumulation to support resistance to drought stress in cotton and increase the relative reduction of the cotton yield under drought conditions.

### The profound impact of BRs on global protein abundance in *pag1* is consistent with the observed phenotypes

Proteomics is a powerful tool for studying plant stress responses, as proteomic comparisons could facilitate the identification of key genes and regulatory mechanisms involved in drought tolerance. In this study, we analyzed differentially abundant proteins in *pag1* and CCRI24 roots, both under normal conditions and after PEG6000 treatment. A total of 2100 differentially abundant proteins were found in *pag1* and CCRI24 samples under both control and PEG6000-treated conditions, and 1105 proteins were revealed to exhibit low abundance in *pag1* roots compared with CCRI24 roots (Fig. 6a, c). Moreover, many stress-related genes were down-regulated in the *pag1* mutant (Table 3). These results suggest that BRs can regulate a large set of proteins, both under normal conditions and upon PEG6000 treatment. In addition, BR deficiency can reduce the protein expression of many well-known stress-responsive genes (Table 3), which may make cotton more sensitive to drought stress.

Through a detailed analysis of differentially abundant proteins between *pag1* and CCRI24 under normal conditions and after PEG6000 treatment, we found that proteins involved in carbon metabolism, starch and sucrose metabolism, carbon fixation in photosynthetic organisms and photosynthesis showed significant reductions in *pag1* plants (Additional file 3: Table S2), although we used the roots for proteomic analysis. These results suggest that BR effect on these proteins, which are mainly involved in photosynthesis, are far-reaching and extend to different organs. It has been reported that BRs can enhance the photosynthesis rate, and can increase seed yield in *B. napus* [15, 24]. Interestingly, our results showed a similar expression pattern of the above proteins in *pag1* (Additional files 3: Table S2), which may partially explain the decreased photosynthetic rate and cotton yield observed in this BR-deficient mutant.

**Fig. 7:**
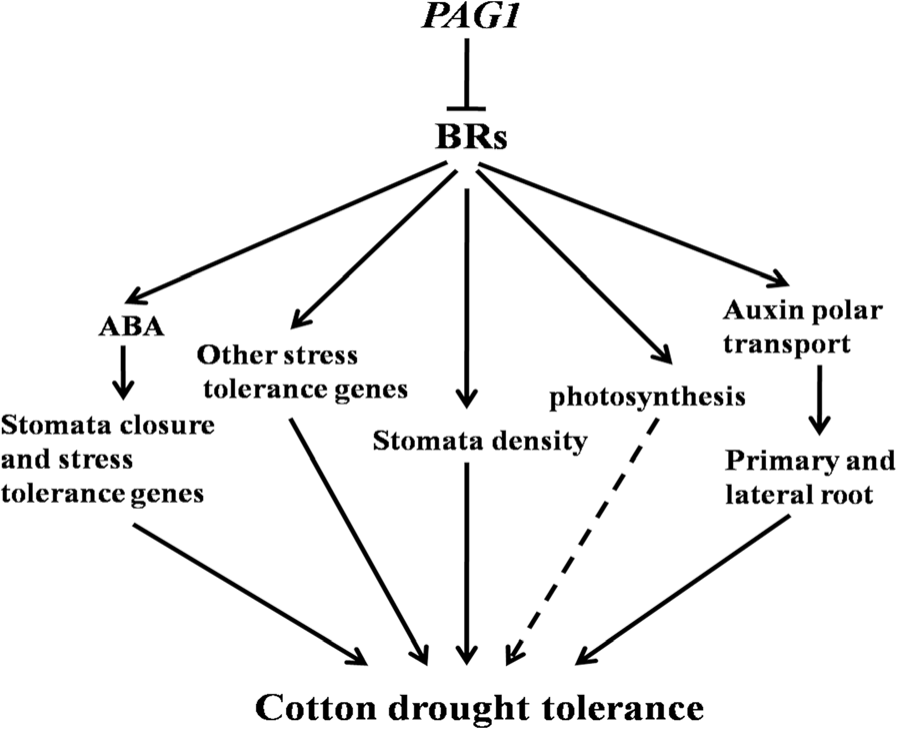
Hypothetical model of *PAG1* and BR regulation of cotton drought tolerance. *PAG1* inhibits endogenous BRs, which promotes ABA accumulation. ABA regulates stomata closure and stress tolerance genes to enhance drought tolerance. BRs regulate auxin polar transport to promote primary and lateral root development and, in turn, enhance drought tolerance. BRs also affect drought tolerance by regulating other stress tolerance genes (ABA independent genes), stomata density and photosynthesis

Moreover, many proteins involved in the ABA signal pathway, auxin signal pathway and root development were also found to be reduced in *pag1* plants through proteomic analysis (Additional file 2: Table S1, Additional file 4: Table S3, Additional file 5: Table S4), and these proteomic results were consistent with the results of the present study (Figs. 3e, 4). Therefore, BR may function upstream of these factors and act as a master integrator of drought tolerance (Fig. 7). Thus, BR deficiency causes a variety of factors to induce cotton sensitivity to drought stress, and the genes involved in BR metabolism can serve as markers to identify plant drought resistance.

## Conclusions

In the present study, we reported that BR deficiency results in cotton more sensitive to drought stress. Further study indicated many aspects such as root growth affected by auxin, stomata development, stomata aperture regulated by ABA, photosynthesis and starch accumulation, and with many stress-related genes were responsible for BR mutant, *pag1*, sensitivity to drought stress. This study demonstrates that BRs play important roles on drought tolerance of cotton and provides an important basis for understanding drought resistance mechanisms regulated by BRs. Overall, our study will provide a vital theoretical basis for genetic improvement and creation of excellent cotton germplasm resources related to drought resistance that regulated by BRs.

## Methods

### Plant materials and growth conditions

For our study, seeds of CCRI24 and *pag1* were obtained from the Institute of Cotton Research of the Chinese Academy of Agricultural Sciences. To examine the resistance of *pag1* to drought stress, seeds from the *pag1* mutant and CCRI24 control plants were delinted, germinated and maintained in pot culture under natural conditions from May to July (2015) in Anyang, Henan Province, China (average daylight length: 14 h, average temperature: 27.7 °C day/17.7 °C nigh). When the cotton seedlings are fifty days old, we don’t water them until the difference in drought phenotypes occurs between *pag1* and CCRI24 plants, then rewatered the cotton seedlings and the number of survival plants was recorded one week later for calculating the survival rate of *pag1* and CCRI24 plants. Treated plants were photographed using a camera (Canon, EOS 70D) to document their phenotypes. Three biological replicates consisting of 10 plants were analyzed for each group.

For seedling hydroculture experiments, *pag1* and CCRI24 seeds were germinated on wet filter paper placed vertically in 1000-ml beakers with 300 ml of water. The beakers were kept in an incubator at a constant temperature of 29 °C under a 16-h light/8-h dark photoperiod. After four days, the germinated cotton seedlings were transferred to cotton nutrient solution (Hoagland’s solution) for growth until the trefoil stage [50, 51]. In addition, approximately 200 *pag1* seedlings were transplanted into cotton nutrient solution containing 24-*epi*-BL at a final concentration of 10 nM, and the shoots were sprayed twice a day with 10 μM 24-*epi*-BL. To further analyze the *pag1* response to drought stress, *pag1* seedlings, *pag1* seedlings treated with 24-*epi*-BL and CCRI24 seedlings were removed from the nutrient solution at the trefoil stage and treated with 6% PEG6000 (drought simulation) for 5 days. Thereafter, the treated seedlings were transferred to normal nutrient solution for another 7 days to analyze the survival rate, and the phenotypes were photographed. Five biological replicates of 8 plants in each experimental group were included in this experiment. To calculate primary root length and lateral root numbers, the roots of 15 plants from each group at the trefoil stage cultivated in nutrient solution were used for analysis. To analyze the fresh root weight or whole-plant weight at the trefoil stage in *pag1* plants, *pag1* plants treated with 24-*epi*-BL and CCRI24 plants, their damp roots were dried with tissue paper to remove moisture, followed by weighing. Thereafter, the roots or whole plants were placed in a paper bag in an electric drying oven (WGLL-230BE) at 65 °C for one week, and the dry weights were then calculated for the roots or whole plants. In these experiments, 15 roots or whole plants were used for the analysis.

### Measurement of stomata density and apertures

To measure stomata density, the top fourth leaves of one-month-old cotton plants were harvested, cut to similar-sized small pieces and placed into 50 ml centrifuge tubes containing 5 ml of reagent I (a mixture of anhydrous ethanol and glacial acetic acid; 9:1, v/v). The reagent was replaced with fresh reagent I after 3 h, and the tubes were incubated in the dark for total of 6 h at 22 °C. After reagent I was decanted, the tubes were rinsed five times each with 90 and 70% ethanol. Then, 5 ml of reagent II, containing glycerin, distilled water and anhydrous chloral (1 ml:1 ml:8 g), was added to the centrifuge tubes, and the samples were stored in the dark for 48 h at 22 °C. Finally, slides of the treated leaf samples were observed and photographed using an Olympus BX51 microscope. For statistical analysis of stomata density, three leaves were sampled from each plant from the *pag1*, *pag1* treated with 24-*epi*-BL and CCRI24, and the stomata were counted under three random microscopic views for each leaf. A room temperature of 22 °C was which the experiment carried out.

Furthermore, to measure the stomata aperture of *pag1* plants, 24-*epi*-BL-treated *pag1* plants and CCRI24 plants, the top fourth leaves of one-month-old plants were detached and placed in a modified stomata-opening buffer (50 mM KCl, 10 mM CaCl2, 10 mM MES, pH 6.15) for 2 h exposed to light according to previous report [52], to open the stomata. Then, ABA was added to the stomata-opening buffer at a final concentration of 10 μM, and the samples were incubated for an additional 2 h while exposed to light [53]. For the 24-*epi*-BL-treated *pag1* samples, 24-*epi*-BL was also added to the stomata-opening buffer at a final concentration of 1 μM. The lower leaf epidermis was removed using transparent scotch tape and transferred to slides. Finally, the stomata were observed and photographed with an Olympus BX51 microscope, and 30 stomata apertures were examined for each treatment. The entire experiment was carried out at a room temperature of 22 °C.

### Hormone measurements

To measure the levels of BRs (BL and CS), approximately 5 g of leaf tissue was collected from one-month-old *pag1* and CCRI24 plants grown under normal conditions in the open field. BRs were quantified by Wuhan Greensword Creation Technology Co., Ltd. as described previously [54]. Three biological replicates were analyzed for this experiment.

For ABA content analysis of the *pag1*, 24-*epi*-BL-treated *pag1* and CCRI24 plants, *pag1* and CCRI24 seeds were sown in pots containing a soil mixture (field soil: nutritional soil: vermiculite = 3:1:1). Approximately 5 g of leaf tissue was harvested from these plants at one month of age, after they had been grown under normal conditions or in the absence of water for one week. ABA levels were determined by Zoonbio Biotechnology Co., Ltd. as described by You et al. [55]. Three biological replicates from each plant group were analyzed.

### Measurement of photosynthetic rates

The photosynthesis rate was measured using a portable photosynthesis system (Li-Cor LI-6400, USA) from 9 to 11 AM on the top fourth leaves of two-month-old cotton plants from the *pag1*, 24-*epi*-BL-treated *pag1* and CCRI24 groups grown in the field. The photosynthetic measurements were taken at a constant photosynthetic photon flux density of 1000 mmol (photon) m^− 2^ s^− 1^. Twenty plants in each group were analyzed.

### Starch staining experiment

To analyze starch accumulation in *pag1*, the top fourth leaves were harvested from two-month-old cotton plants of the *pag1*, 24-*epi*-BL-treated *pag1* and CCRI24 groups, and 5-mm leaf pieces were cut out using a hole puncher and immersed in 95% ethanol in 50-ml centrifuge tubes for 24 h in the dark. The ethanol was then decanted, and the leaf pieces were stained with 2% iodine-potassium iodide (I_2_-KI) for half an hour [56]. Then, the leaf pieces were photographed using a stereomicroscope (Motic, Moticam Pro 285A). Three biological replicates from each group were used for this experiment.

### Transmission electron microscopy (TEM) analysis

To observe starch accumulation in the chloroplasts of *pag1*, 24-*epi*-BL-treated *pag1* and CCRI24 plants, the top fourth leaves were harvested from two-month-old cotton plants, and ultrathin tissue sections were obtained. Ultrathin sectioning was performed according to a previous report [57]. Finally, the sections were observed and photographed using a New Bio-TEM H-7500 transmission electron microscope (HITACHI, Japan). This experiment applied three biological replicates from each group.

### RNA extraction and cDNA preparation

To extract total RNA, plant samples were quickly collected and frozen in liquid nitrogen, and RNA extraction and first-strand cDNA synthesis were performed as described previously [50]. The cDNA was used as a template in the subsequent real-time quantitative RT-PCR (qRT-PCR) analyses.

### Quantitative RT-PCR

qRT-PCR was performed to assess the relative transcript levels of polar auxin transport carrier genes and stress-related genes expressed in the roots of the *pag1*, 24-*epi*-BL-treated *pag1* and CCRI24 plants at the trefoil stage after treatment with 6% PEG6000 for different lengths of time (0, 12, 24 and 36 h). For qRT-PCR, the reactions were prepared and conducted as previously described [50]. Each amplification reaction was performed in triplicate, and three biological replicates were quantified. The *G. hirsutum* histone-3 gene (*GhHIS3*) was used as an internal control, and the results were calculated using the 2^−ΔΔCT^ method.

### Field trials

To evaluate the agronomic traits of *pag1*, 24-*epi*-BL-treated *pag1* and CCRI24 plants, field trials were carried out from May to October (2015) in Anyang, Henan Province, China, under normal and drought conditions in the field. To evaluate the agronomic traits of plants under drought condition, *pag1* and CCRI24 seeds were grown in the field under a water proof shed. This field trial involved three replicate plots of approximately 5 m^2^ each. Each plot comprised one row of CCRI24 plants and two rows of *pag1* plants; one row of *pag1* plants was sprayed with 10 μM 24-*epi*-BL twice a day beginning on the day the *pag1* seedlings broke through the soil. Each plot included approximately 60 plants, with 30 cm inter-row spacing and 20 cm interplant spacing. The field was watered only once at sowing time, and during the remainder of the growing season, the cotton plants in each plot were grown under natural drought conditions. A duplicate set of plants was planted in another field with full irrigation to evaluate the agronomic traits of *pag1*, 24-*epi*-BL-treated *pag1* and CCRI24 plants under normal conditions. Ten plants were randomly selected from each experimental group in each plot for agronomic trait and cotton yield analyses.

### iTRAQ-based proteomic assay

To analyze the differentially abundant proteins in *pag1* and CCRI24 plants, cotton seedlings were cultivated using the hydroculture method described above and treated with 6% PEG6000 for different lengths of time (0, 12, 24 and 36 h) at the trefoil stage. Next, 10 g root samples were collected, and proteomic analysis was performed by Shanghai Applied Protein Technology Co., Ltd. as described in a previous report [58]. Three biological replicates were analyzed for this experiment. Changes in protein abundance between *pag1* and CCRI24 plants at four different treatment time points were determined to be significant if the abundance ratio was ≥1.2 and the *P*-value determined by Student’s t test was < 0.05. Gene ontology (GO) enrichment of the differentially abundant proteins was determined according to the methods used by Dong et al. [59]. A heatmap was generated using R software (version 3.4.1), and a Venn diagram was drawn using the Venny 2.1.0 [60].

## Additional files

Additional file 1: **Figure S1.** Expression patterns of ten genes determined from the proteomic results for *pag1* and CCRI24 under normal conditions and at 12 h, 24 h and 36 h after PEG treatment. P, *pag1*; C, CCRI24; 0, 12, 24 and 36 represent the trefoil-stage roots of *pag1* and CCRI24 after treatment with 6% PEG6000 for 0 h, 12 h, 24 h and 36 h. (DOCX 2427 kb)

Additional file 2: **Table S1.** Down-regulated genes involved in the ABA signal pathway in the *pag1* mutant. (DOCX 14 kb)

Additional file 3: **Table S2.** Down-regulated genes involved in carbon metabolism, starch and sucrose metabolism, carbon fixation in photosynthetic organisms and photosynthesis in the *pag1* mutant. (DOCX 14 kb)

Additional file 4: **Table S3.** Down-regulated genes involved in the auxin signal pathway in the *pag1* mutant. (DOCX 13 kb)

Additional file 5: **Table S4.** Down-regulated genes involved in root development in the *pag1* mutant. (DOCX 14 kb)

Additional file 6: **Table S5.** Specific qRT-PCR primers used for analysis of the expression patterns of stress-related genes. (DOCX 13 kb)

## Abbreviations

24-*epi*-BL: 24-*epi*brassinolide
ABA: Abscisic acid
BL: Brassinolide
BR: Brassinosteroid
BRs: Brassinosteroids
CCRI24: Chinese cotton research institution 24
CS: Castasterone
GO: Gene ontology
*pag1*: *pagoda1*
PEG: Polyethylene glycol
PME: Pectin methylesterase
SRS3: The third substrate recognition sites
