# Supplementary material for: BR deficiency causes increased sensitivity to drought and yield penalty in cotton
Source: BMC Plant Biol. 2019 May 28;19:220. doi: 10.1186/s12870-019-1832-9 (PMC6537406; doi:10.1186/s12870-019-1832-9)
Supplement: Supplementary file 1 — Figure S1. Expression patterns of ten genes determined from the proteomic results for pag1 and CCRI24 under normal conditions and at 12 h, 24 h and 36 h after PEG treatment. P, pag1; C, CCRI24; 0, 12, 24 and 36 represent the trefoil-stage roots of pag1 and CCRI24 after treatment with 6% PEG6000 for 0 h, 12 h, 24 h and 36 h. (DOCX 2427 kb) [file 12870_2019_1832_MOESM1_ESM.docx]

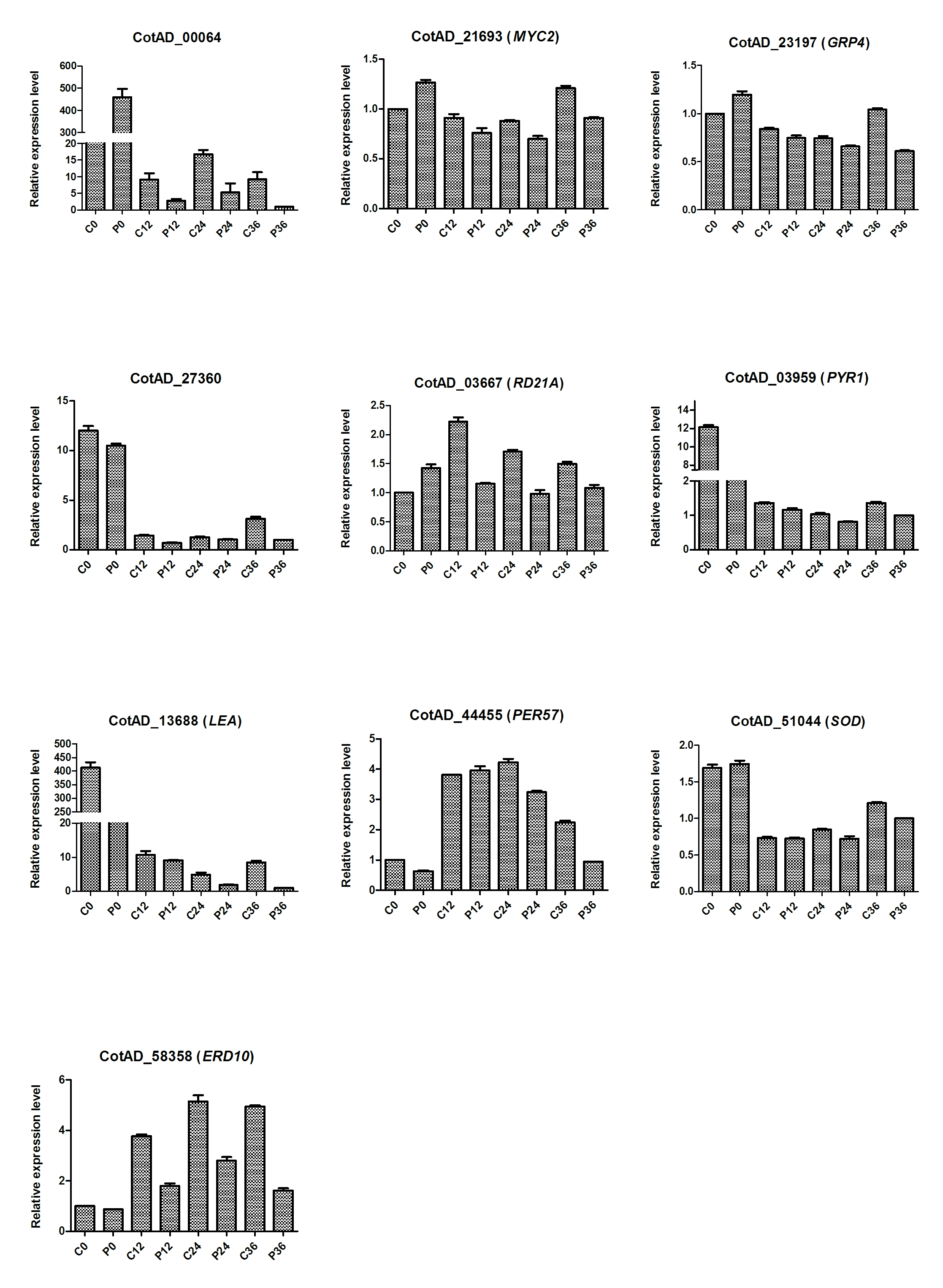


**Figure S1.** The expression patterns of ten genes obtained from proteomic results in *pag1* and CCRI24 under normal condition and at 12 h, 24 h and 36 h after PEG treatment . P, *pag1*; C, CCRI24; 0, 12, 24, 36 represent the trefoil stage roots of *pag1* and CCRI24 after treatment of 0 h, 12 h, 24 h, 36 h by 6% PEG6000.
