# Supplementary material for: BR deficiency causes increased sensitivity to drought and yield penalty in cotton
Source: BMC Plant Biol. 2019 May 28;19:220. doi: 10.1186/s12870-019-1832-9 (PMC6537406; doi:10.1186/s12870-019-1832-9)
Supplement: Supplementary file 2 — Table S1. Down-regulated genes involved in the ABA signal pathway in the pag1 mutant. (DOCX 14 kb) [file 12870_2019_1832_MOESM2_ESM.docx]

**Table S1.** The down-regulated genes that involved in ABA signal pathway in the *pag1* mutant.

| Protein ID | Annotation | E-Value |
| --- | --- | --- |
| CotAD_57059 | abscisic acid-deficient 4 | 3.01E-168 |
| CotAD_54244 | farnesylcysteine lyase | 0 |
| CotAD_30026 | ruBisCO large subunit-binding protein subunit alpha | 0 |
| CotAD_35404 | UBP1-associated protein 2c | 0 |
| CotAD_21693 | MYC2, bHLHprotein isoform 1 | 0 |
| CotAD_65255 | ammonium transporter 1 member 1-like | 0 |
| CotAD_03959 | abscisic acid receptor PYL1-like | 1.34E-141 |
| CotAD_04202 | transcription factor HY5 | 9.64E-87 |
| CotAD_06884 | vesicle-associated membrane protein 713 | 2.03E-145 |
| CotAD_58684 | protein aspartic protease in guard cell 2-like | 0 |
| CotAD_00064 | protein aspartic protease in guard cell 2-like | 0 |
| CotAD_40692 | protein aspartic protease in guard cell 2-like | 0 |
| CotAD_35386 | peroxygenase 4 -like protein | 4.91E-144 |
| CotAD_27297 | protein aspartic protease in guard cell 2-like | 0 |
| CotAD_01695 | RNA-binding family isoform 1 | 0 |
| CotAD_32224 | catalase isozyme 1 | 0 |
