# Supplementary material for: BR deficiency causes increased sensitivity to drought and yield penalty in cotton
Source: BMC Plant Biol. 2019 May 28;19:220. doi: 10.1186/s12870-019-1832-9 (PMC6537406; doi:10.1186/s12870-019-1832-9)
Supplement: Supplementary file 3 — Table S2. Down-regulated genes involved in carbon metabolism, starch and sucrose metabolism, carbon fixation in photosynthetic organisms and photosynthesis in the pag1 mutant. (DOCX 14 kb) [file 12870_2019_1832_MOESM3_ESM.docx]

**Table S2.** The down-regulated genes that involved in carbon metabolism, starch and sucrose metabolism, carbon fixation in photosynthetic organisms and photosynthesis in the *pag1* mutant.

| Protein ID | Annotation | E-Value |
| --- | --- | --- |
| CotAD_74086 | phosphoglycerate mutase family protein | 0 |
| CotAD_32447 | 6-phosphogluconate decarboxylating | 0 |
| CotAD_47709 | 6-phosphogluconate decarboxylating | 0 |
| CotAD_58191 | NADH-dependent malic enzyme | 0 |
| CotAD_40995 | NADH-dependent glyceraldehyde-3-phosphate dehydrogenase-like | 0 |
| CotAD_03898 | malate | 0 |
| CotAD_36193 | methylenetetrahydrofolate reductase 2-like | 0 |
| CotAD_07946 | polygalacturonase | 0 |
| CotAD_39793 | UDP-glucuronic acid decarboxylase 2-like | 0 |
| CotAD_58191 | NADH-dependent malic enzyme | 0 |
| CotAD_03898 | malate | 0 |
| CotAD_29976 | ROOT FNR 1 isoform 1 | 0 |
| CotAD_54975 | oxygen-evolving enhancer protein | 1.27E-63 |
| CotAD_01588 | ROOT FNR 1 isoform 1 | 0 |
