# Supplementary material for: BR deficiency causes increased sensitivity to drought and yield penalty in cotton
Source: BMC Plant Biol. 2019 May 28;19:220. doi: 10.1186/s12870-019-1832-9 (PMC6537406; doi:10.1186/s12870-019-1832-9)
Supplement: Supplementary file 4 — Table S3. Down-regulated genes involved in the auxin signal pathway in the pag1 mutant. (DOCX 13 kb) [file 12870_2019_1832_MOESM4_ESM.docx]

**Table S3.** The down-regulated genes that involved in auxin signal pathway in the *pag1* mutant.

| Protein ID | Annotation | E-Value |
| --- | --- | --- |
| CotAD_50420 | auxin-induced in root cultures 12 -like protein | 0 |
| CotAD_54840 | probable NAD(P)H dehydrogenase FQR1-like 2 | 1.11E-164 |
| CotAD_04805 | probable acyl-activating enzyme peroxisomal isoform x1 | 0 |
| CotAD_20424 | UDP-l-rhamnose synthase | 0 |
| CotAD_32617 | protein auxin response 4 | 0 |
| CotAD_11502 | chalcone synthase 1 | 0 |
| CotAD_24302 | benzoquinone reductase | 1.13E-122 |
| CotAD_63438 | probable NAD(P)H dehydrogenase FQR1-like 2 | 1.18E-162 |
| CotAD_06195 | xyloglucan endotransglucosylase hydrolase protein 22-like | 4.66E-178 |
| CotAD_64484 | tryptophan synthase alpha chain-like | 0 |
