# Supplementary material for: BR deficiency causes increased sensitivity to drought and yield penalty in cotton
Source: BMC Plant Biol. 2019 May 28;19:220. doi: 10.1186/s12870-019-1832-9 (PMC6537406; doi:10.1186/s12870-019-1832-9)
Supplement: Supplementary file 5 — Table S4. Down-regulated genes involved in root development in the pag1 mutant. (DOCX 14 kb) [file 12870_2019_1832_MOESM5_ESM.docx]

**Table S4.** The down-regulated genes that involved in root development in the *pag1* mutant.

| Protein ID | Annotation | E-Value |
| --- | --- | --- |
| CotAD_49973 | protein root hair defective 3-like isoform x1 | 0 |
| CotAD_59195 | SMAD/FHA domain-containing protein isoform 3 | 0 |
| CotAD_65255 | ammonium transporter 1 member 1-like | 0 |
| CotAD_50420 | auxin-induced in root cultures 12-like protein | 0 |
| CotAD_07982 | two-component response regulator ARR1-like protein | 0 |
| CotAD_08987 | nucleolin like 2 isoform partial | 4.26E-165 |
| CotAD_02287 | glycosyl hydrolase family 38 protein isoform 1 | 0 |
| CotAD_38140 | nucleolin 1-like isoform x3 | 0 |
| CotAD_12042 | nucleolin 1-like isoform x3 | 5.15E-177 |
| CotAD_41504 | translationally controlled tumor protein | 3.03E-89 |
| CotAD_33106 | germin-like protein subfamily 2 member 4 | 7.22E-123 |
| CotAD_06738 | profilin 1 isoform 1 | 3.17E-80 |
| CotAD_25212 | profilin 1 isoform 1 | 2.98E-80 |
| CotAD_55827 | mannosyl-oligosaccharide 1,2-alpha-mannosidase MNS1-like | 0 |
| CotAD_20970 | biotin lipoyl attachment domain-containing protein isoform 1 | 0 |
| CotAD_19756 | monocopper oxidase-like protein SKU5 | 0 |
| CotAD_11370 | monocopper oxidase-like protein SKU5 | 0 |
