# Supplementary material for: BR deficiency causes increased sensitivity to drought and yield penalty in cotton
Source: BMC Plant Biol. 2019 May 28;19:220. doi: 10.1186/s12870-019-1832-9 (PMC6537406; doi:10.1186/s12870-019-1832-9)
Supplement: Supplementary file 6 — Table S5. Specific qRT-PCR primers used for analysis of the expression patterns of stress-related genes. (DOCX 13 kb) [file 12870_2019_1832_MOESM6_ESM.docx]

**Table S5.** The specific qRT-PCR primers used for analysis the expression patterns of stress-related genes.

| **Primer name** | **Sense primer** | **Antisense primer** |
| --- | --- | --- |
| *CotAD_00064* | ATGGCGACTATGATTGTACCA | GGTGAAGAATATGGTGAAAGG |
| *CotAD_21693* | TCGGATTCTAAACCTACGCC | GCAACAACTCCATGCTCTCA |
| *CotAD_23197* | TAGAGGAAGCTGAGAAAGGAA | CGGCCATAGGAATTATAGCC |
| *CotAD_27360* | TGGCGAACTCTAAGCTTACC | TCTGAGACATCCGTGCCTAA |
| *CotAD_03667* | CCTACCGGCAGAAAACTGC | CCGTTAAAAATACCCGATGCA |
| *CotAD_03959* | AATGCCTGACCCACCACCT | ATAAGGAAGAACACTGACCTG |
| *CotAD_13688* | ATGGAAGCTAGCAATGTGCAA | AAACTCTTTCCTGTCCAGTAC |
| *CotAD_44455* | AGTTGTCCTCGAGCTGAATC | TTCTTTCTCTGATCGGCTTTG |
| *CotAD_51044* | TGTCGACAAACAGATTCCTCT | TCACCCTTGCAGACCGATAA |
| *CotAD_58358* | AGCCAGTAGTCCAGCCAGA | CTTTGACTCCCCTTCGTGATG |
| *GhHis3* | GCCAAGCGTGTCACAATTATG | ACATCACATTGAACCTACCACTACC |
